# Cases of Fracture of Cranium and Dislocation of Ulna

**Published:** 1845-07-01

**Authors:** Darwin Colvin


### Cases of fracture of Cranium and dislocation of Ulna.

Communicated in a letter to Prof. Hamilton, by DARWIN COLVIN, M. D.

PROF. HAMILTON: — CLYDE, April 3, 1845.

*Dear Sir*—I send you a case of fracture of the Cranium, with wound of the Median Meningeal Artery, producing extravasation and death. The circumstances were these: — A father and son while attending our town meeting became intoxicated, and in consequence of the son's ill treatment of his father, the old gentleman threw a piece of board and it struck him (his son) about the squamous portion of the temporal bone. *He did not fall*, but seemed to be rather unconscious for a minute, when he recovered, and was about the streets for an hour or so, seeming to be as well as usual, trading horses. &c. In about two hours he began to manifest some symptoms of compression, vomiting, inability to stand, &c. He was put into the waggon, carried home, which was about six o'clock in the evening; did not speak during the short time he lived, *and no medical advice was called until five the following morning*. I was then sent for, but he died before I arrived, and no examination was allowed at that time. Our people, however, together with myself, suspecting that his death was in consequence of the blow, obtained a coroner's inquest, and upon examination, I found a fracture of the squamous portion, together with a wound of the Median Meningeal Artery as it lay enclosed in its bony canal. There was a very large coagulum resting upon the left hemisphere. My testimony was that he came to his death by a blow inflicted by his father.

My father and myself have had another very interesting case viz. of luxtion of the upper end of the Ulna, *without fracture of the Olecranon or Coronoid*. A gentleman in descending the stairs of his barn fell, and luxated the upper end of the Ulna inward and outward. When we saw him we found the olecranon process presenting anteriorly, and the coronoid a little more inward than backward; in fact the bone had slipped around and rested on the anterior surface of the inner condyle! We could detect no fracture, and, as it has terminated, there was none. We found great difficulty in reducing it, it requiring three assistants. The integument was so tense over the coronoid that ecchymosis had taken place, amounting, in fact, almost to a compound dislocation. The elbow from side to side measured some six inches. I applied a straight splint for a short time. The inflammation at first was very severe and the swelling great. After it began to subside I had him use motion, which he now does, and the joint seems to be very perfect. He cannot extend it quite so far as the other, but I think this is in consequence of the inflammation and swelling not having entirely subsided, the extension increasing as the inflammation subsides. We hope for its successful recovery.

I am yours, &c., DARWIN COLVIN.

